# Isolation and functional analyses of *PvFAD2* and *PvFAD3* involved in the biosynthesis of polyunsaturated fatty acids from Sacha Inchi (*Plukenetia volubilis*)

**DOI:** 10.7717/peerj.9169

**Published:** 2020-05-26

**Authors:** Tianquan Yang, Xiaojuan Wang, Tingnan Dong, Wei Xu, Aizhong Liu

**Affiliations:** 1 Germplasm Bank of Wild Species, Kunming Institute of Botany, Chinese Academy of Sciences, Kunming, Yunnan, China; 2 Department of Resources and Environmental Engineering, Henan University of Engineering, Zhengzhou, Henan, China; 3 Key Laboratory for Forest Resource Conservation and Utilization in the Southwest Mountains of China, Ministry of Education, Southwest Forestry University, Kunming, China; 4 Department of Economic Plants and Biotechnology, and Yunnan Key Laboratory for Wild Plant Resources, Kunming Institute of Botany, Chinese Academy of Sciences, Kunming, Yunnan, China

**Keywords:** Fatty acid desaturase, Polyunsaturated fatty acid, Sacha Inchi, Vegetable oils, Heterologous expression

## Abstract

The development of *ω*-3 fatty acid-rich vegetable oils is essential to enrich the production of functional foods. Sacha Inchi (*Plukenetia volubilis* L.) is a unique oilseed crop with much potential. Its seeds contain rich polyunsaturated fatty acids (PUFAs), especially linoleic acid (LA, C18:2) and *α*-linolenic acid (ALA, C18:3). Endoplasmic reticulum -located *ω*-6 and *ω*-3 fatty acid desaturases (FAD) are responsible for the biosynthesis of LA and ALA, respectively, in plant seeds. Here, we isolated two full-length *FAD* genes from Sacha Inchi, named *PvFAD2* and *PvFAD3*, which encoded predicted amino acid residues of 384 and 379 in protein, respectively. Protein sequence and subcellular localization analysis revealed that they were located in the endoplasmic reticulum (ER). Heterologous expression in *Saccharomyces cerevisiae* confirmed that PvFAD2 and PvFAD3 could catalyze LA and ALA synthesis, respectively. The stability and catalytic efficiency of the PvFAD3 protein may be closely related to temperature. In transgenic tobacco, using seed-specific expression promoters, PvFAD2 and PvFAD3 significantly promotes the production of LA (from 68% to 70.5%) and ALA (from 0.7% to 3.1%) in seed oil. These results show that PvFAD2 and PvFAD3 do, indeed, function as crucial enzymes for PUFAs biosynthesis, and provide a key gene source for the sustainable production of lipids with tailored fatty acid compositions via genetic engineering in other oil crops.

## Introduction

Sacha Inchi (*Plukenetia volubilis* L., Euphorbiaceae), which bears star-shaped fruit capsules, known as mountain peanut, Inca nut or Inca Inchi, is native to the Amazon region of South America, including parts of Peru and northwestern Brazil (Hamaker et al., 1992; Gillespie, 2007). Since its seeds contain rich nutritive materials such as edible oil (45% of dry weight), protein (27%), tocopherols (137 mg/100 g) and phytosterols (75 mg/100 g) (Cai et al., 2011; Chirinos et al., 2013; Chirinos et al., 2015), Sacha Inchi seeds (and its derivatives) have been used in traditional cuisine for hundreds of years by indigenous people. Importantly, most of the fatty acids from its seed oils are highly unsaturated, containing about 77.5–84.4% polyunsaturated fatty acids (PUFAs) and 8.4–13.2% monounsaturated fatty acids, while only a small proportion of its seed oil is saturated, comprising only 7.9–9.1% (Chirinos et al., 2013; Maurer et al., 2012; Gutiérrez, Rosada & Jiménez, 2011; Follegatti-Romero et al., 2009). Notably, in Sacha Inchi seed oils, there is an abundance of *ω*-3 *α*-Linolenic acid (ALA), accounting for about 46–50%, followed by the *ω*-6 linoleic acid (LA), accounting for about 33–36% (Chirinos et al., 2015; Wang & Liu, 2014). Studies have demonstrated that the proportion of *ω*-6 and *ω*-3 fatty acids in edible oils is critical in preparing functional foods to treat or prevent cardiometabolic disease, heart disease, hypertension and diabetes (Patterson et al., 2012; Lee et al., 2009; Wijendran & Hayes, 2004). The ratio of *ω*-6 to *ω*-3 fatty acids in Sacha Inchi seed oils ranges from 0.83 to1.09, which is considered to be an optimal proportion of functional oils for human health (Simopoulos, 2011; Simopoulos, 2008). However, the physiological and molecular mechanism that underlie the accumulation of the high content of ALA in Sacha Inchi seeds largely remain uncertain. Dissecting the molecular mechanism behind ALA biosynthesis in Sacha Inchi seeds and identifying key genes responsible for controlling or regulating the biosynthesis of ALA would facilitate the use of genetic engineering in crop breeding to produce lipids with tailored fatty acid compositions as well as the provision of healthy functional oils.

Fatty acid desaturases (FADs) play a pivotal role in the conversion of saturated FAs into unsaturated FAs via catalyzing the formation of a double bond between two carbon atoms (C-C) at a specific location of FAs. In general, the first step of PUFAs biosynthesis begins with the de novo formation of acyl-chains in the plastid by the fatty acid synthase (FAS) complex in plastid, generating primary palmitoyl-ACP (C16-ACP) and Stearory-ACP (C18-ACP). Then Stearory-ACP can be desaturated by a soluble stearoyl-ACP desaturase (SAD) to form oleoyl-ACP (C18:1-ACP). Acyl chain elongation terminated by acyl-ACP thioesterases (FAT), and the released oleic acid (C18:1) is then incorporated into glycerolipid biosynthesis and further desaturated into LA and ALA by two fatty acid desaturase enzymes: FAD2 (catalyzing Δ12/*ω*-6 fatty acid) and FAD3 (catalyzing Δ15/*ω*-3 fatty acid) in endoplasmic reticulum (ER). Although both *FAD2* and *FAD3* genes have been extensively identified in oilseed plants, such as tree peony, olive, *Jatropha curcas,* soybean, sunflower, sesame and *Brassica napus* (Yin et al., 2018; Wu et al., 2013; Li et al., 2007; Rolletschek et al., 2007; Hernández, Sicardo & Martínez-Rivas, 2016; Hernández, Mancha & Martínez-Rivas, 2005; Jin et al., 2001; Reed, Schafer & Covello, 2000), the potential molecular mechanism for the extensive variation in different fatty acid proportions in vegetable oils remains largely unknown. Based on transcriptomic data, gene sequence similarity analysis and expression investigation, we identified *PvFAD2* and *PvFAD3* and found their seed-specific expression in developing Sacha Inchi seeds (Wang et al., 2012; Wang & Liu, 2014; Hu et al., 2018), but their functions in controlling PUFAs biosynthesis were not tested.

In this study, we cloned the full-length cDNA sequences of two fatty acid desaturase genes (*PvFAD2* and *PvFAD3*) and characterized their functions in controlling PUFAs biosynthesis by heterologous transformation in yeast (*Saccharomyces cerevisiae*) and tobacco. This study not only characterized the functions of the PvFAD2 and PvFAD3 in controlling PUFA biosynthesis, but also provided critical insights into understanding the physiological and molecular mechanism of the higher content of *α*-Linolenic acid in Sacha Inchi seed oils, supporting the utilization of functional oil foods.

## Materials & Methods

### Total RNA extraction and cDNA preparation

Sacha Inchi plants were grown under natural climate conditions at the Xishuangbanna Tropical Botanical Garden (XTBG), Chinese Academy of Sciences (CAS), Yunnan, China. The developing seeds were collected 45 days after pollination (DAP) from a three-year-old individual (at this stage of seed development, the lipids are accumulated rapidly). The developing seed tissues were dissected and stored in a freezer at −80 °C until use. Total RNA was extracted using RNAprep Pure Plant Kit (DP432; TianGen) following the manufacturer’s protocol. The cDNA was synthesized from total RNA using a PrimerScript 1st Strand cDNA Synthesis Kit (Takara, Dalian, China) according to the manufacturer’s instructions.

### Isolation of two fatty acid desaturase genes

To obtain the full-length nucleotide sequence of *PvFAD2* and *PvFAD3*, we downloaded transcriptome sequencing reads from Short Read Archive (accession number: SRP101395) produced from different tissues of Sacha Inchi (Hu et al., 2018) and assembled them using the Trinity software (Grabherr et al., 2011). The coding sequence (CDS) of *AtFAD2* (AT3G12120) and *AtFAD3* (AT2G29980) from Arabidopsis were respectively used as a query to BLAST against the assembled transcripts database using the BLAST software (version 2.2.24+: https://blast.ncbi.nlm.nih.gov/Blast.cgi?PAGE_TYPE=BlastDocs&DOC_TYPE=Download). Based on amino acid sequence alignment and functional domain analysis, we found the *PvFAD2* transcript contained a full-length coding sequence. The *PvFAD2* gene was further comfirmed by high fidelity PCR using TransStart FastPfu DNA Polymerase (TransGen, Beijing, China) with the specific primers, PvFAD2-F and PvFAD2-R (Table S1). To obtain the full-length of *PvFAD3* nucleotide sequences, we adopted a 5′ and 3′ RACE (rapid amplification of cDNA ends) method using a SMART RACE cDNA Amplification Kit (Clontech, Palo Alto, CA) according to the manufacturer’s protocol. All used primer sequences are listed in Table S1. The final PCR products with the full-length *PvFAD3* nucleotide were purified and cloned into the pEASY-Blunt cloning Vector (TransGen, Beijing, China) for subsequent sequencing to confirm the full-length *PvFAD3* CDS. The obtained full-length CDS of both PvFAD2 and PvFAD3 were submitted to GenBank with accession numbers MK121677 (PvFAD2) and MK121679 (PvFAD3).

### Sequence alignment and phylogenetic analysis

Homologous proteins of both PvFAD2 and PvFAD3 were retrieved from NCBI by BLASTP program (https://blast.ncbi.nlm.nih.gov/Blast.cgi), and resulting sequence information were listed in Table S2. For the phylogenetic analysis of PvFAD2 and PvFAD3, we included the plastid-located Δ12/*ω*-6 (FAD6) and Δ15/*ω*-3(FAD7 and FAD8) fatty acid desaturases from representative plants (see Table S2). Multiple sequence alignments were performed by the DNAMAN program. Phylogenetic analysis was conducted using MEGA (version 5.0) with the Neighbor-Joining criteria (Tamura et al., 2011). Branch support of the phylogenetic tree was estimated on the base of 10,000 bootstrap replicates of the data.

### Subcellular localization of PvFAD2 and PvFAD3

The full-length CDS of *PvFAD2* and *PvFAD3* were inserted into the binary vector *pCambia35S::GFP* to respectively generate *35S::GFP-PvFAD2* and *35S::GFP-PvFAD3* constructs. Subsequently, the constructed plasmids were transformed into the *Agrobacterium tumefa ciens* (EHA105). Positive clones were selected on an LB plate supplemented with rifampicin (50 mg/L) and kanamycin (50 mg/L), and cultured in liquid LB medium containing appropriate antibiotics at 28 °C. After centrifugation, the pellets were resuspended in the infiltration buffer (10 mM MES, 200 µM acetosyringgone and 10 mM MgCl_2_, PH5.6) until the OD600 of 0.8 for the final cell density. After 2 hours standing at room temperature, the constructs were delivered into the lower epidermis of 10-week-old tobacco leaves by agro-infiltration. Fluorescence was observed at 2–4 days post-transfection using the confocal system (Olympus, Fluoview FV1000).

### Heterologous transformation in yeast (*Saccharomyces cerevisiae*)

To dissect the function of *PvFAD2* and *PvFAD3* in yeast, we constructed yeast expression vectors using homologous recombination strategy. Briefly, the full-length CDS was amplified by PCR using Phanta Max Super-Fidelity DNA polymerase (Vazyme, Nanjing, China). The primers used in this step were listed in Table S1. The PCR products were purified and then ligated into P426-GAP vector (digested with *BamH I* and *EcoR I*) using ClonExpress Entry One Step Cloning Kit (Vazyme, Nanjing, China). Finally, the plasmid harbored the *PvFAD2* or *PvFAD3* was transformed into *S. cerevisiae* (INVSc1) using Frozen-EZ Yeast Transformation II Kit (Zymo, USA). An empty vector was also transformed into *S. cerevisiae* as a negative control. Positive clones were obtained via selection on plates of SD medium lacking uracil at 30 °C. Recombinant strains of *S. cerevisiae* were then transferred into lipid SD medium lacking uracil, and shaken at 30 °C/230 rpm. To test PvFAD3 function, we exogenously added methyl linoleate (Sigma, USA) into media for the supply of C18:2 fatty acid substrates. When cells reached the stationary phase, cells were collected for subsequent analysis.

### Heterologous transformation in tobacco

To inspect the functions of *PvFAD2* and *PvFAD3* in plants, the two genes were heterologously transformed into tobacco, and specifically expressed in developing seeds. The full-length CDS of *PvFAD2* and *PvFAD3* were constructed into the binary vector pCambia2300 under the control of the seed-specific *Napin* promoter, and named *Napin::PvFAD2* and *Napin::PvFAD3.* These vectors carry the NptII gene encoding neomycine phosphotransferase conferring kanamycin resistance. Constructs were next transformed into tobacco (*Nicotiana tabacum* L. cv. Honghua dajinyuan) using the Agrobacteria-mediated tobacco leaf-disk transformation method (Horsch et al., 1985). Transgenic plants (T_0_) were selected on shoot induction and root induction medium containing 100 mg/L kanamycin. To test whether the targeted genes were successfully transformed into the selected individuals, the genomic DNA was isolated from young leaves of T_0_ plants and PCR amplification was performed to confirm the presence of the targeted gene with specific primers. The expression levels of transformed FAD genes in transgenic plants were tested using quantitative real-time PCR (qRT-PCR). Total RNAs were isolated from developing seeds using RNAprep Pure Plant Kit (TianGen, DP432) and reversely transcripted using PrimerScrip™ RT reagent Kit with gDNA Erases (Takara, China). qRT-PCR was performed on the CFX96 machine (Bio-Rad, Hercules, USA) according to the following program: pre-cycling at 94 °C for 30 s followed by 42 cycles of 94 °C for 5 s, 56 °C for 15 s and 72 °C for 10 s. The *NtActin* gene was used as an internal control. All primers used in this study were listed in Table S1. The matured seeds of T_0_ plants were harvested for the next analysis.

### Lipid extraction and fatty acid composition analysis

Total lipids were extracted from transgenic yeast and transgenic mature seeds, and fatty acid methyl esters (FAMEs) were prepared according to our previous description (Xu et al., 2014; Yang et al., 2016). To analyse the lipid composition of the yeast, 20 mL of different yeast strain cells at the stationary growth phase were collected and dissolved in 4 M HCl. After boiling for 10 min, total lipids were extracted using 500 µL hexane/isopropanol (3:2, v/v). Tobacco seeds from each line were homogenised in 500 µL hexane/isopropanol (3:2, v/v). Then the total lipids were dissolved in 500 µL chloroform and transmethylated with 2 mL of methanol containing 5% H_2_SO_4_ (v/v) and then heated at 85 °C for 90 min. Finally, FAMEs were subjected to Gas Chromatograph (Agilent 6890N) and Gas Chromatography-Mass Spectrometer (Agilent 7890/5975C) for fatty acid species analysis.

## Results

### Isolation and sequence analysis of two FA desaturase genes

**Figure 1:**
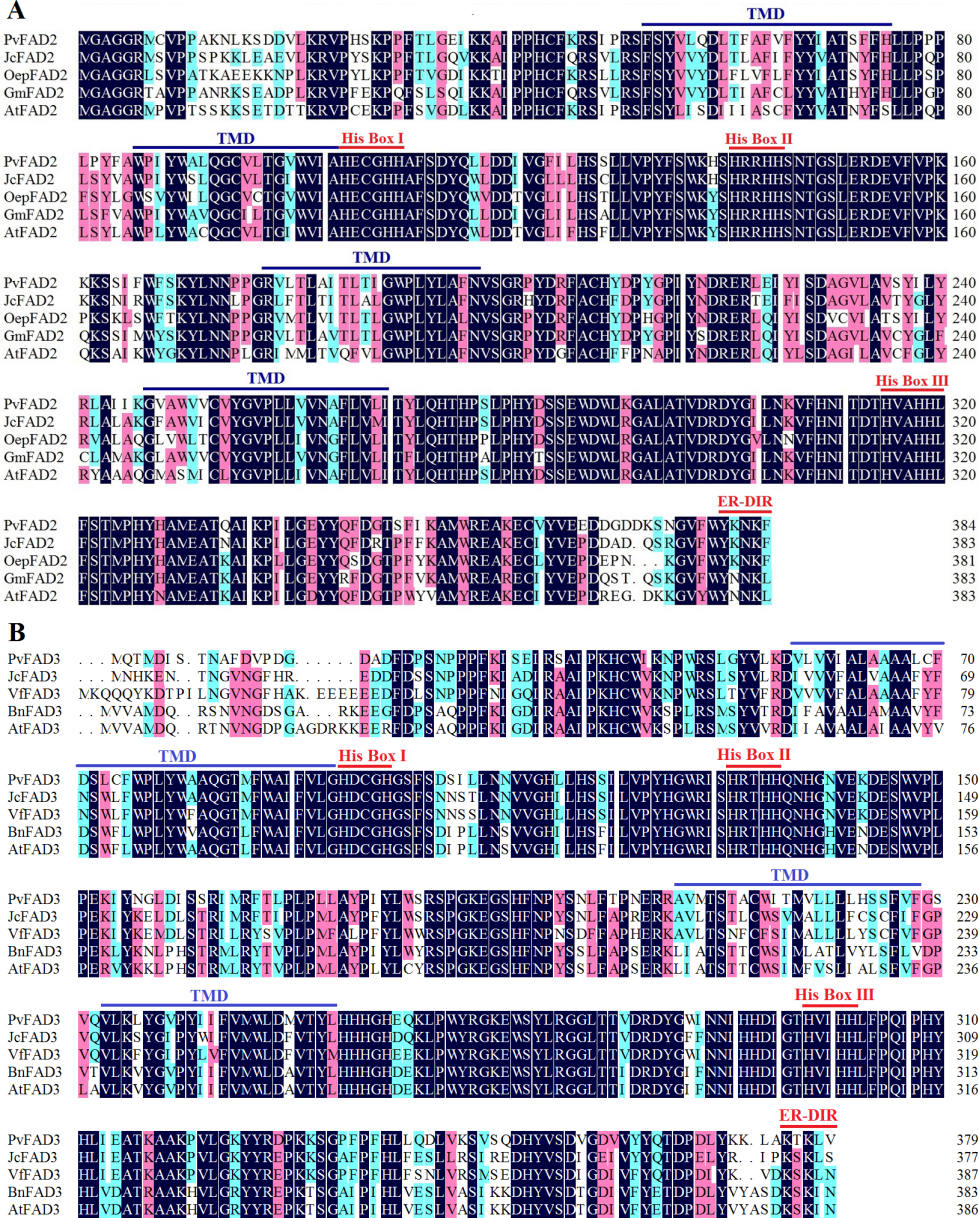
Comparison of the amino acid sequences of the PvFAD2 and PvFAD3 from Sacha Inchi with other plants. (A) Comparison of PvFAD2 with JcFAD2 (*Jatropha curcas*), OeFAD2 (*Olea europaea*), GmFAD2 (*Glycine max*) and AtFAD2 (*Arabidopsis thaliana*). (B) Comparison of PvFAD3 with JcFAD3 (*J. curcas*), VfFAD3 (*Vernicia fordii*), BnFAD3 (*Brassica napus*) and AtFAD3 (*A. thaliana*). The conserved histidine residues (His Box), ER signal sequences (ER-DIR), and the potential transmembrane domains (TMDs) were indicated. The color marks show the conserved degree of amino acid residues at different levels (the dark denotes 100%, the pink denotes ≥75%, and blue denotes ≥50%).

The full-length of the coding sequences of *PvFAD2* and *PvFAD3* were 1152 bp (encoding 384 amino acid residues with a calculated molecular mass of 44.19kDa) and 1137 bp (encoding 379 amino acid residues with a calculated molecular mass of 43.53 kDa), respectively. To characterize the structural features of PvFAD2 and PvFAD3 proteins, we performed multiple sequence alignments together with other functionally characterized homologs from plants, such as *Jatropha curcas*, *Olea europaea*, *Glycine max*, *Vernicia fordii*, *Brassica napus* and *Arabidopsis thaliana* (Fig. 1) (Wu et al., 2013; Hernández, Mancha & Martínez-Rivas, 2005; Li et al., 2007; O’Quin et al., 2010; Reed, Schafer & Covello, 2000; Botella et al., 2016). The result showed that all FAD2 proteins contained four transmembrane domains (TMDs) and three histidine boxes (HXXXH, HXXHH and HXXHH) essential for desaturase activity (Fig. 1A) (Shanklin, Whittle & Fox, 1994). Similarly, all FAD3 proteins contained four TMDs and eight conserved histidine residues in three histidine boxes (Fig. 1B). In addition, in the C-terminal FAD2 and FAD3 contained a ER-DIR motif known as an ER location signal (Dehghan Nayeri & Yarizade, 2014; McCartney et al., 2004). It should be noted that the N-terminal sequences of FAD3 proteins exhibited high variation, notwithstanding the high similarities to other regions (Fig. 1B). Collectively, the alignment of the amino acid sequences of FAD showed that PvFAD2 and PvFAD3 shared the three conserved histidine rich motifs with other known functional homologs, indicative of the proper encoding of full-length cDNA of both isolated PvFAD2 and PvFAD3.

### Phylogenetic analysis of FA desaturases

**Figure 2:**
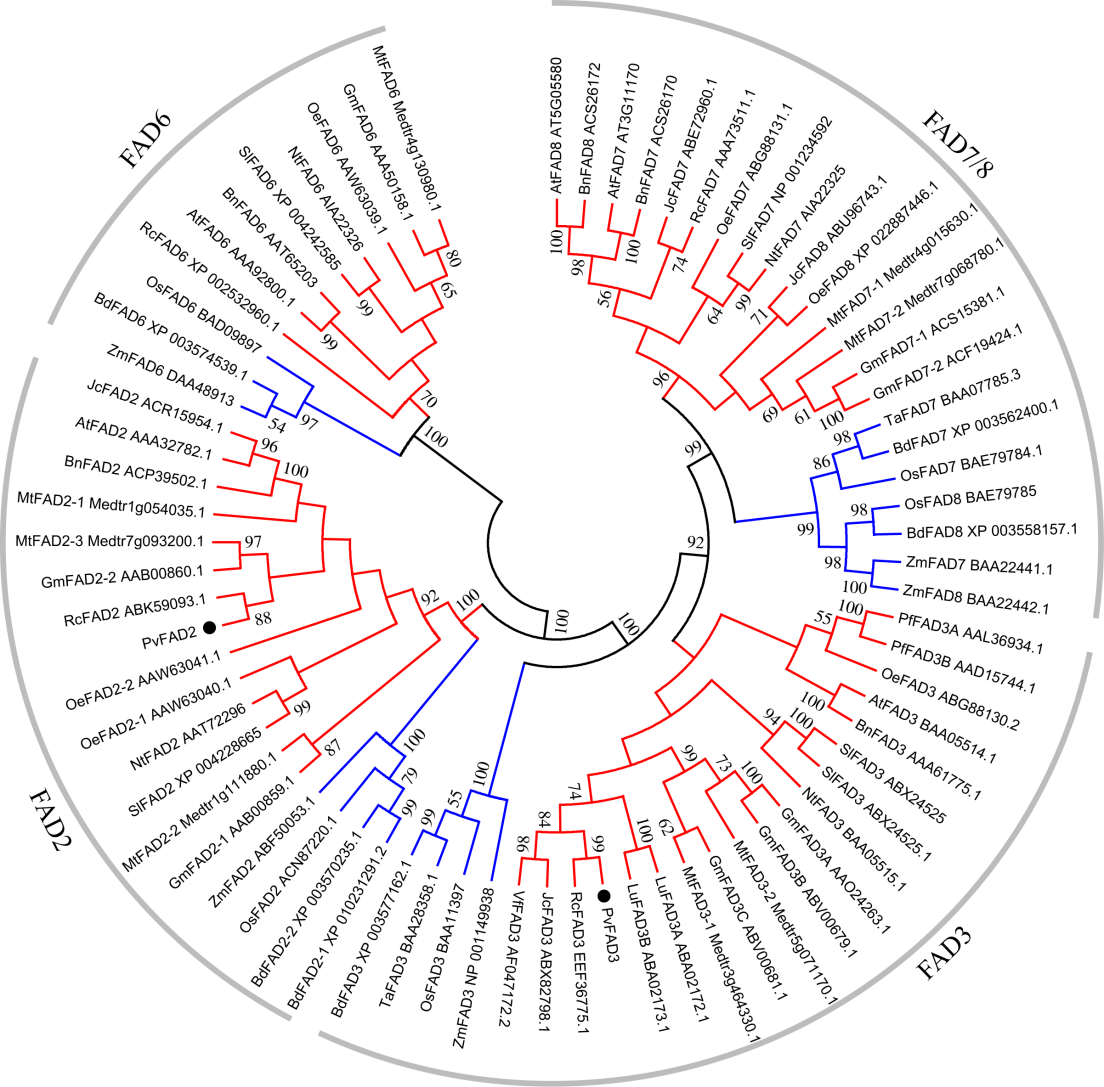
A Phylogenetic tree of *FAD* genes obtained from Sacha Inchi and other plants. Based on the Neighbor-Joining (NJ) criteria using MEGA5 program; bootstrap values (≥50) for each branch were indicated by numbers on lines. The red and blue branches denote the FAD proteins from dicotyledon and monocotyledon, respectively.

To elucidate the phylogenetic relationship of plant membrane fatty acid desaturase, an unrooted neighbor-joining (NJ) tree was constructed by the deduced amino acid sequences of FA desaturases from diverse flowering species, including plastidial *ω*-3 (FAD7/8), ER-location *ω*-3 (FAD3), plastidial *ω*-6 (FAD6) and ER-location *ω*-6 (FAD2) (Table S2; Fig. 2). The topology of this tree clearly showed that FA desaturases appear divided into four well-resolved monophyletic groups with well-supported bootstrap values (Fig. 2), suggesting the conserved role of FA desaturases in each group. These groups consist of the FAD6 group catalyzing the formation of Δ12/*ω*-6 fatty acids in plastid; the FAD2 group responsible for the formation of Δ12/*ω*-6 fatty acids in ER, the FAD7/8 group that is Δ15/*ω*-3 desaturases in plastid, and the FAD3 group that is Δ15/*ω*-3 desaturases in ER. Moreover, in each group, most dicot members formed a group relative to the monocots in the same clade, which may stem from the divergent evolution of dicots and monocots. Plant plastid-localized FAD6 showed a close relationship with ER-location FAD2, while plastid-localized FAD7/8 were clearly clustered with ER-location FAD3 (Fig. 2), suggesting a common evolutionary origin of Δ12/*ω*-6 desaturases or Δ15/*ω*-3 desaturases located in ER and plastids (Fig. 2). PvFAD2 and PvFAD3 were clustered with ER-located Δ12/*ω*-6 and Δ15/*ω*-3 desaturases, respectively, implying that they have a conserved functional role in catalyzing the formation of *ω*-6 or *ω*-3 fatty acids by operating in ER.

### Subcellular localization of PvFAD2 and PvFAD3 proteins

**Figure 3:**
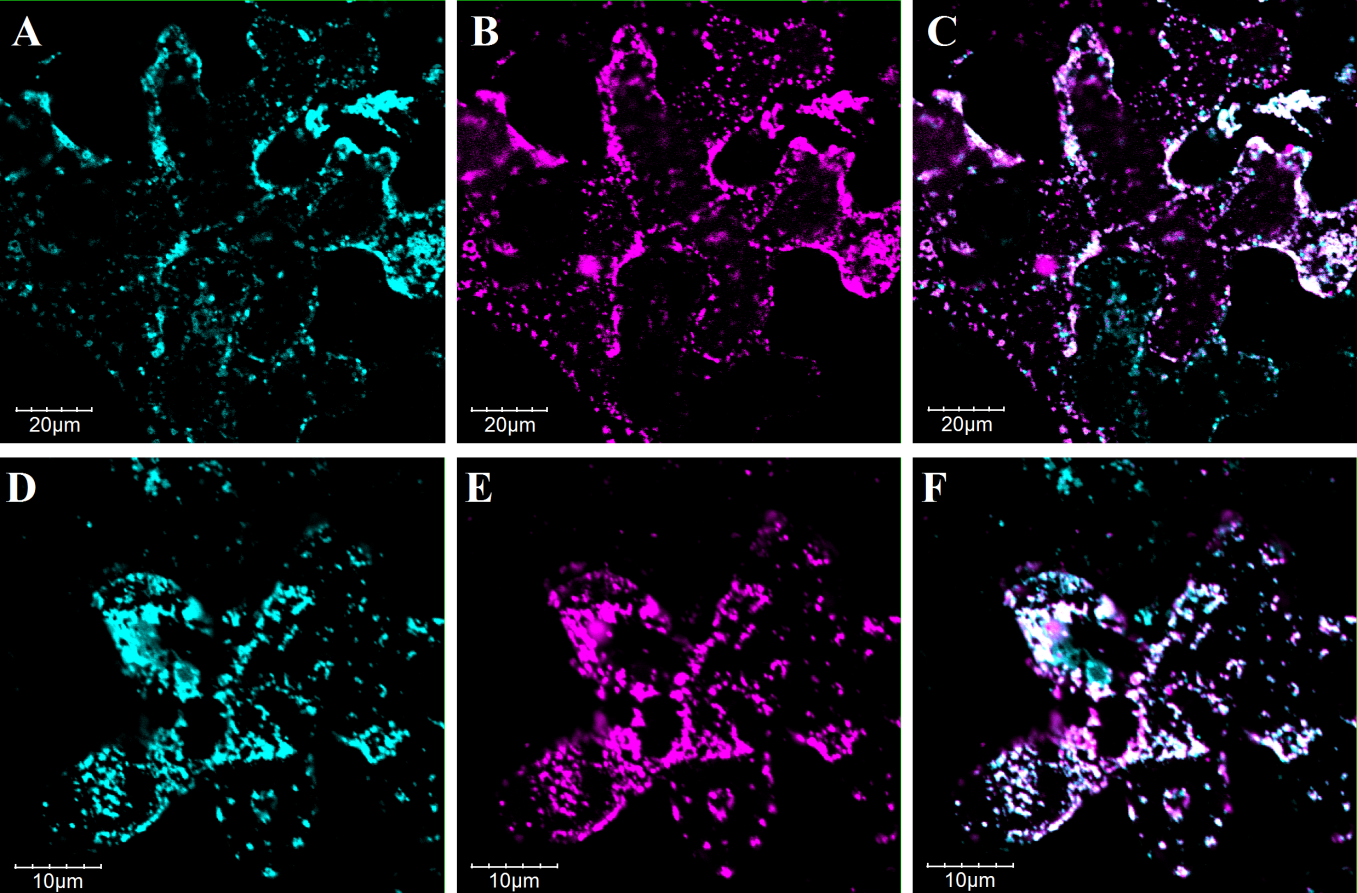
Subcellular localization analysis of PvFAD2 and PvFAD3 in tobacco epidermal cells. (A–C) Colocalization of 35S::GFP-PvFAD2 with ER-mCherry (CD3-939). (D–F) Colocalization of 35S::GFP-PvFAD3 with ER-mCherry. The cyan signals in A and D obtained with confocal microscopy indicated fusion protein of GFP-PvFAD2 and GFP-PvFAD3. The magenta signals in B and E exhibited the mCherry (ER-marker protein). The merged of GFP and mCherry fluorescent signals is indicated in overlay images C and F.

The intracellular desaturation of fatty acid chains occurs widely in different compartments such as plastid for synthesizing the desaturated membrane lipids and ER for synthesizing the desaturated storage lipids (Tocher, Leaver & Hodgson, 1998). To further inspect the subcellular location of PvFAD2 and PvFAD3, that is, where they function in desaturating fatty acids, we transiently expressed their proteins fused with GFP in epidermal cells of tobacco leaves. As shown in Fig. 3, under confocal microscopy a distinct subcellular co-localization of PvFAD2 and PvFAD3 protein with a ER membrane marker CD3-959-mCherry were observed, indicating that PvFAD2 and PvFAD3 function to produce desaturated fatty acids in ER.

### Functional analysis the PvFAD2 and PvFAD3 in yeast (*S. cerevisiae*)

The two full-length cDNAs *PvFAD2* and *PvFAD3* were cloned into the yeast expression vector P426 for the detection of their enzymatic activities by investigating their changes in their lipid content and fatty acid composition. As shown in Table 1A, the fatty acid compositions obtained from the *S. cerevisiae* with the empty vector (P426), grown under normal growth condition (30 °C), mainly included palmitic acid (C16:0), palmitoleic acid (C16:1), stearic acid (C18:0) and oleic acids (C18:1). When we heterologously expressed *PvFAD2* in *S. cerevisiae* cells, we found substantial changes in FA species, resulting in two new peaks corresponding to hexadecadienoic acid (C16:2) and linoleic acid (C18:2, LA), respectively (also see Fig. S1A). Subsequently, the proportion of C16:1 or C18:1 fatty acids was significantly reduced in the lipids obtained from transformants, from 38.9% to 36.27% and from 35.44% to 24.34% respectively, while other FA species did not display a distinct change (Table 1A). These results suggested that PvFAD2 can desaturate both C16:1 and C18:1 FA and produce the C16:2 and C18:2.

**Table 1:** Fatty acid composition of *S. cerevisiae* overexpressing two FAD from Sacha Inchi.

A. PvFAD2		C16:0	C16:1	C16:2	C18:0	C18:1	C18:2
30 °C	P426	18.35 ± 0.57	38.90 ± 1.25^a^	–	7.31 ± 0.54	35.44 ± 0.84^a^	–
	P426-PvFAD2	17.99 ± 0.68	36.27 ± 1.27^b^	4.25 ± 0.14	7.70 ± 0.43	24.34 ± 1.17^b^	9.34 ± 0.42
20 °C	P426	16.37 ± 1.12	41.29 ± 2.01^a^	–	6.77 ± 0.69	35.57 ± 1.51^a^	–
	P426-PvFAD2	16.15 ± 0.75	32.58 ± 1.21^b^	3.53 ± 0.18	8.12 ± 0.35	29.44 ± 0.71^b^	10.18 ± 0.55

B. PvFAD3		C16:0	C16:1	C18:0	C18:1	C18:2 (Feeding)	C18:3
30 °C	P426	17.65 ± 1.07^a^	8.02 ± 0.76	6.45 ± 0.37	8.21 ± 0.56	59.43 ± 1.59	–
	P426-PvFAD3	19.94 ± 0.38^b^	9.28 ± 2.70	7.33 ± 0.34	11.40 ± 2.73	50.47 ± 5.78	1.58 ± 0.41^a^
20 °C	P426	16.86 ± 0.95	14.50 ± 2.45	4.95 ± 0.30^a^	10.62 ± 1.69^a^	52.94 ± 4.49	–
	P426-PvFAD3	19.34 ± 1.98	18.55 ± 3.24	6.08 ± 0.33^b^	16.56 ± 1.91^b^	37.12 ± 4.91	2.35 ± 0.32^b^

**Notes.**

The mean value was obtained from four biological replicates. The significance was tested by one-way ANOVA analysis. The letter a and b indicated the significant difference (*p* < 0.05) between two groups.

Usually, ER-location *ω*-3 desaturase can directly catalyze the C18:2 into C18:3 by introducing third double bonds into the Δ15/*ω*-3 position of fatty acids. In order to investigate the function of PvFAD3, we expressed *PvFAD3* in *S. cerevisiae* cells with exogenous C18:2 in the media, due to the yeast cells lack of C18:2 fatty acids (see Fig. S1B) (Reed, Schafer & Covello, 2000; Vrinten et al., 2005; Yin et al., 2018). As a result, under normal growth conditions (30 °C), yeast cells carrying *PvFAD3* in culture, supplemented with C18:2, produced an additional peak, which we identified as C18:3 by GC-MS, while in yeast cells carrying the empty vector P426, the C18:3 was not detected (Fig. S1B; Table 1B). The amount of C18:3 in expressed *PvFAD3* yeast cells reached by 1.58% of total lipids. Meanwhile, we noted that the yeast cells could easily absorb the exogenous C18:2, up to 59.43% of total lipids, but the content of C16:1 (8.02%) and C18:1 (8.21%) was significantly reduced as compared to the wild yeast cells (38.9% for C16:1 and 35.44% for C18:1), suggesting that the absorption of C18:2 followed a substantial inhibition of the biosynthesis pathway C16:1 and C18:1. In summary, this clearly showed that *PvFAD2* and *PvFAD3* genes from Sacha Inchi encode functional FA desaturase.

Many studies have found that the enzyme stability and catalytic efficiency of FAD2 and FAD3 in plants is often influenced by temperature (Li et al., 2007; O’Quin et al., 2010; Wang & Liu, 2014). To test this factor, we cultured yeast cells carrying the *PvFAD2* or *PvFAD3* gene under low (20 °C) and high temperature (30 °C) to compare changes in the proportion of FA composition. As shown in Table 1, when PvFAD2 transformed yeast cells were cultured under low temperature conditions, the content of C16:2 and C18:2 was changed but not significantly, decreasing from 4.25% to 3.53% for C16:2, and increasing from 9.34% to 10.18% for C18:2 relative to the 30 °C growth condition. In PvFAD3 transformed yeast cells, low temperatures promoted the biosynthesis of C18:3 in which the amount of C18:3 significantly increased by 48.7% from 1.58% to 2.35% when compared with high temperatures. These results showed that changes in temperature did not affect the activity of PvFAD2, whereas PvFAD3 protein were sensitive to temperature changes.

### Functional analysis of PvFAD2 and PvFAD3 in tobacco seeds

**Figure 4:**
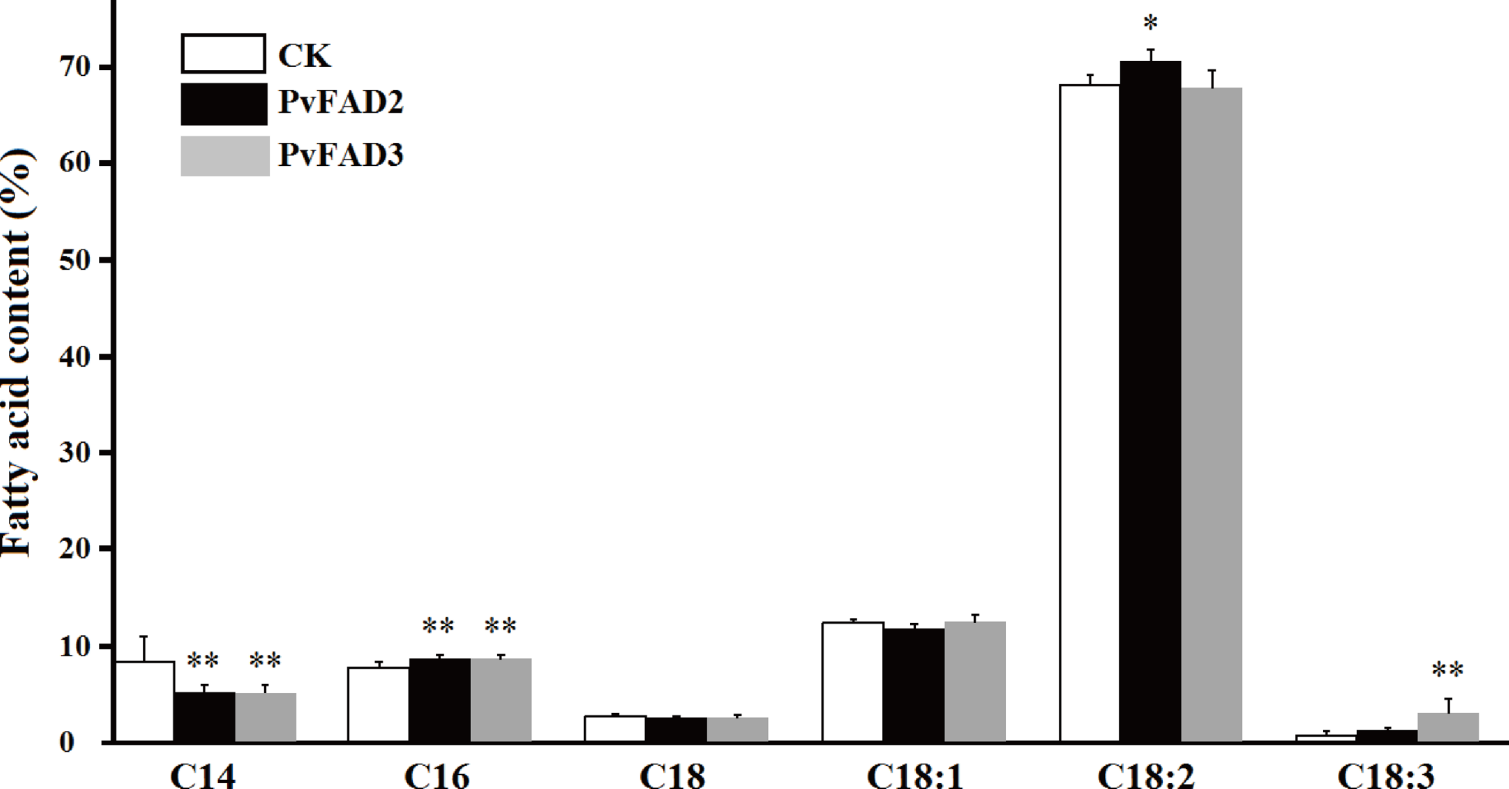
Changes of fatty acid composition in transgenic tobacco seed oils. Comparison the contents of different fatty acids between the transformants (*PvFAD2* and *PvFAD3*) and the wide type (CK), as a control. Five independent transformants of *PvFAD2* and six independent transformants of PvFAD3 were tested with the significance (*T*-test, *P < 0.05, **P < 0.01).

To uncover the functional role of *PvFAD2* and *PvFAD3* in plant seeds, these two genes with the seed-specific promoters were transformed into tobacco via the leaf-disk transformation method. Transformants were selected by kanamycin, and further confirmed by PCR analysis as described in the ‘Materials and Methods’ section. In total, five independent transgenic lines of *PvFAD2* and six independent transgenic plants of *PvFAD3* were selected and cultivated in the greenhouse. Compared to wild type plants, the *PvFAD2* and *PvFAD3* were highly expressed in transgenic lines (Fig. S2). During plant growth, we observed no obvious phenotypic differences between transgenic and wild-type plants. At maturity, seeds from the T_0_ generation of transgenic tobacco were collected to measure seed weight, oil content and fatty acid composition. As a result, we found no obvious change in seed weight and oil content in transgenic lines relative to wide-type plants, but the proportion of different fatty acids was significantly changed (Fig. 4). In transgenic PvFAD2 seeds, C18:2 content reached 70.5% of total lipids, and was significantly higher than that for wide-type plants (67.0% of total lipids), although the reduction in C18:1 content from 12.4% to 11.8% was not significant. We also observed that the proportion of C16:0 increased from 7.7% to 8.7%, whereas the proportion of C14:0 substantially decreased from 8.3% to 5.2%. In transgenic PvFAD3 seeds, the proportion of C18:3 was markedly increased by about 3.4 fold, from 0.7% in wild type to 3.1% in *PvFAD3* expressing lines, while C18:2 content, a substrate of C18:3, was unchanged compared to wild-type lines (see Fig. 4). Similarly, the amount of C14:0 and C16:0 were substantially changed, with a significant decrease for C14:0 from 8.3 % to 5.1% and increase for C16:0 from 7.7% to 8.7% (Fig. 4). This result from transgenic tobacco seeds clearly showed that PvFAD2 and PvFAD3 make substantial contributions to the production of C18:2 and C18:3, respectively.

## Discussion

The exploration of PUFAs (especially ALA-rich) vegetable oils is essential for the production of foods with diverse functions. Although studies have documented that the production of PUFAs in seed oil is usually controlled by several ER-located FAD genes such as FAD2 and FAD3, and many FAD2 and FAD3 genes have been extensively identified in oilseed plants, relatively few FAD genes, for any given plant, have been functionally examined (Dar et al., 2017). The potential molecular mechanisms for the extensive variation in different fatty acid proportions in vegetable oils remains largely unknown. As mentioned above, Sacha Inchi is a promising oilseed crop that contains critical nutrients and is an indispensable compound for developing functional foods due to its seed oils, which are rich in LA and ALA-helpful for protection against several diseases (Lee et al., 2016). For resource discovery or breeding of function food plants, it is necessary to understand the potential physiological and molecular mechanisms behind the high proportion of LA and ALA found in Sacha Inchi seed oils.

In this study, we isolated two full-length *PvFAD2* and *PvFAD3*, which were seed-specifically expressed, and associated with seed oil accumulation (Wang et al., 2012; Hu et al., 2018). Our results strongly suggest that their functions might be directly involved in regulating the biosynthesis of PUFAs in Sacha Inchi seeds. Multiple sequence alignments showed that both PvFAD2 and PvFAD3 contain typical transmembrane domains (TMDs) and three histidine boxes (His Box). In plants, the production of Δ12/ *ω*-6 fatty acids can be catalyzed by either ER-located FAD2 or plastid-located FAD6, and Δ15/*ω*-3 fatty acids catalyzed by either ER-located FAD3 or plastid-located FAD7/8. Although these two classes of FA desaturases, ER- or plastid-located desaturases can introduce double bonds to the same position as fatty acids, phylogenetic analysis revealed their evolutionary divergence. The PvFAD2 and PvFAD3 that we isolated belong to ER-located desaturases and are clustered with other plant orthologs, suggesting that they are an evolutionarily conserved feature. Subcellular localization experiments confirmed that the PvFAD2 and PvFAD3 desaturases functioned in the endoplasmic reticulum, suggesting that they could be predicted to participate in the biosynthesis of PUFAs in seed oils (storage lipids).

Because of the conserved lipid biosynthesis pathway (Kennedy pathway), the functions of plant FA-modifying enzymes can be tested by heterologous expression in yeast. In particular, *S. cerevisiae* has been developed as a good system to test the function of lipid genes due to its simple fatty acid composition and easy transformation (Yin et al., 2018; Li et al., 2007). Here, heterologous expressions of both *PvFAD2* and *PvFAD3* in *S. cerevisiae* demonstrated that they could encode FA desaturase, catalyzing the biosynthesis of PUFAs. Interestingly, the heterologous expression of *PvFAD2* generated both C18:2 and C16:2 fatty acids, suggesting that PvFAD2 might bifunction at two sites on the carbon chains of fatty acids (using C18:1 and C16:1 as substrates) in yeast. However we did not determine whether the heterologous expression of *PvFAD2* could generate C16:2 fatty acids in transformed tobacoo seeds because we did not detect C16:2 and its substrate C16:1 fatty acids. It is uncertain whether PvFAD2 are able to catalyze C16:1 into C16:2 because of the lack of substrate C16:1 in tobacco seeds. Similarly, the C18:2 content was significantly elevated in transgenic PvFAD2 tobacco seeds, although the amount of C18:1 maintained a relatively constant level. Meanwhile, the significant decrease in C14 and increase in C16 may suggest the promotion of flux from short length chain fatty acid (<C16) to long length fatty acid (C16 and C18) in transgenic plants. Feeding with exogenous C18:2 fatty acids, the heterologous expression of PvFAD3 generated C18:3 fatty acids, meaning that the enzyme PvFAD3 is active in catalyzing biosynthesis of C18:3 fatty acids in this yeast system.

Functional PvFAD2 and PvFAD3 were further confirmed in transgenic tobacco seeds. However, it should be noted that these two FA desaturases exhibited low efficiency for the production of PUFAs in both yeast and tobacco seed systems, producing an undesirably low output amount for their use. In both transformed yeast cells and tobacco seeds, we noted that the substrates C18:1 (for producing C18:2 by PvFAD2) and C18:2 (for producing C18:3 by PvFAD3) should be sufficient to produce C18:2 and C18:3, respectively (Table 1 and Fig. 4), but only a small number of substrates were desaturated. These results suggest that the heterologous expression of single genes (*PvFAD2* or *PvFAD3*) offer a limited contribution to generate PUFAs in both transgenic yeast cells and tobacco seeds. The developing Sacha Inchi seeds accumulate massive PUFAs (in particular the ALA) and oil may require co-function of many genes involved in fatty acid biosynthesis, carbon chain desaturation and triglyceride assembly (Wang et al., 2017). Based on the heterologous expression of single *PvFAD2* or *PvFAD3* in transgenic yeast cells and tobacco seeds, our current study demonstrated their functions in producing C18:2 by PvFAD2 and producing C18:3 by PvFAD3. However, this work cannot explain the potential mechanism underlying the massive accumulation of PUFAs in Sacha Inchi seeds. The expression regulation of *PvFAD2* and *PvFAD3* might be an important clue to understand the potential mechanism for the massive accumulation of PUFAs (in particular ALA) in Sacha Inchi seeds. In addition, the biosynthesis of PUFAs-rich or unusual FA oils often requires some selective acyltransferases which are able to selectively incorporate specific fatty acids into a glycerol skeleton. For instance, studies have found that some diacylglycerol acyltransferases (DGAT) such as JcDGAT2 (identified from *Jatropha curcas*), RcDGAT2 (isolated from castor bean) and VfDGAT2 (isolated from tung tree) are able to selectively incorporate PUFAs-rich or unusual FAs into triacylglycerols (Lunn, Wallis & Browse, 2019; Shockey et al., 2006; Burgal et al., 2008; Li et al., 2010; Li, Yu & Hildebrand, 2010; Xu et al., 2014). However, whether the biosynthesis of ALA-rich oils needs the participation of some selective acyltransferases in Sacha Inchi seeds remains unknown.

Additionally, we found that the thermal stability of PvFAD3 was sensitive to temperature in yeast cells, which is consistent with our previous observations of Sacha Inchi seeds. When Sacha Inchi grew in seasons with low temperatures its seed oils accumulated a higher proportion of ALA compared with when grown in the hot season (Wang & Liu, 2014). Similarly, previous studies had shown that the stability of protein FAD3 was temperature-dependent, such as BnFAD3 (identified from *Brassica napus*) (Dyer et al., 2001). In particular, increasing evidence has shown that FAD3 proteins are extensively regulated at the post-transcriptional level in a temperature-dependent manner, for example, the stability of BnFAD3 and VfFAD3 (isolated from *Vernicia fordii*) proteins are sensitive to the regulation of cis-acting degradation signals within their N terminal (O’Quin et al., 2010). When comparing the amino acid sequences in the N terminal (Fig. 1) we found that they were highly divergent among plants. Thus, it remains unclear how temperature can influence the stability of PvFAD3 protein, resulting in the change of ALA proportions in Sacha Inchi seed oils.

In summary, this study represents the first attempt to identify and functionally characterize two fatty acid desaturase genes, *PvFAD2* and *PvFAD3*. Our results showed that PvFAD2 and PvFAD3 are able to functionally catalyze LA and ALA in both yeast cells and tobacco seeds, which strongly suggests that PvFAD2 and PvFAD3 play a key role in generating PUFAs-rich oils in Sacha Inchi seeds. However, the molecular mechanism underlying the highly-efficient accumulation of ALA in Sacha Inchi seed remains to be determined. Exploring the key genes or regulators involved in controlling the accumulation of PUFAs oils, in particular ALA-rich oils in Sacha Inchi seeds, would provide an inspiring way to discover functional foods, based on ALA-rich oils, by using genetic and metabolic engineering techniques.

## Conclusions

Sacha Inchi is a unique oilseed crop with great potential due to its seeds containing rich polyunsaturated fatty acids (PUFAs), especially linoleic acid (LA) and *α*-linolenic acid (ALA). It is necessary to understand the potential physiological and molecular mechanisms behind the high proportion of LA and ALA found in Sacha Inchi seed oils. This study represents the first attempt to identify and functionally characterize two fatty acid desaturases genes, *PvFAD2* and *PvFAD3* from Sacha Inchi. Our results showed that PvFAD2 and PvFAD3 are able to functionally catalyze LA and ALA in both yeast cells and tobacco seeds, which strongly suggests that PvFAD2 and PvFAD3 play crucial roles in PUFAs biosynthesis, and provide a key gene source for the sustainable production of lipids with tailored fatty acid compositions via genetic engineering in other oil crops.

##  Supplemental Information

10.7717/peerj.9169/supp-1 Figure S1 Gas chromatogram of fatty acid methyl esters from yeast cultures transformed with the P426 vector alone and P426 with PvFAD2 and PvFAD3 (A) PvFAD2 expression in yeast cells. (B) PvFAD3 expression in yeast cells, supplemented with methyl linoleate. The yeast cultures were grown at 30 °C until stationary phase. 33777A2DB

10.7717/peerj.9169/supp-2 Figure S2 Expression level analysis of *PvFAD2* and *PvFAD3* in transgenic tobacco seeds by real time qRT-PCR. Every individual plant was tested for three replicates (A) PvFAD2 expression level in five transformed plants. (B) PvFAD3 expression level in six transformed plants.

10.7717/peerj.9169/supp-3 Table S1 Primers used in this study

10.7717/peerj.9169/supp-4 Table S2 FAD proteins used in sequence alignment and phylogenetic analysis

## Additional Information and Declarations

### DNA Deposition

The following information was supplied regarding the deposition of DNA sequences: The obtained full-length CDS of both PvFAD2 and PvFAD3 are available at GenBank: PvFAD2, MK121677; PvFAD3, MK121679.

### Data Availability

The following information was supplied regarding data availability: The raw measurements are available in the Supplemental Files.
