# Supplementary figures and images for: Isolation and functional analyses of PvFAD2 and PvFAD3 involved in the biosynthesis of polyunsaturated fatty acids from Sacha Inchi (Plukenetia volubilis)
Source: PeerJ. 2020 May 26;8:e9169. doi: 10.7717/peerj.9169 (PMC7315619; doi:10.7717/peerj.9169)

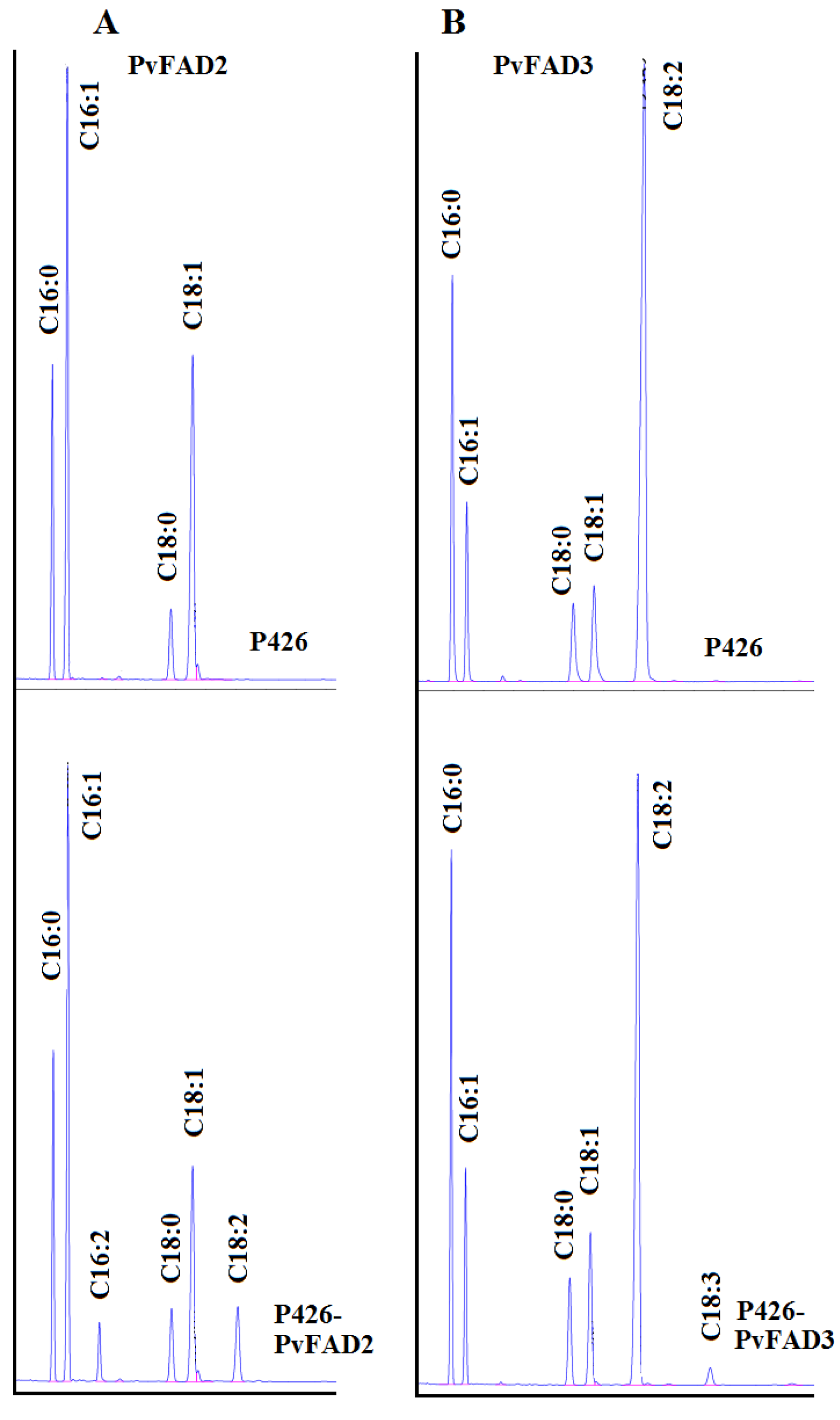

Supplement: Figure S1 — (A) PvFAD2 expression in yeast cells. (B) PvFAD3 expression in yeast cells, supplemented with methyl linoleate. The yeast cultures were grown at 30 °C until stationary phase. 33777A2DB [file peerj-08-9169-s001.tif]

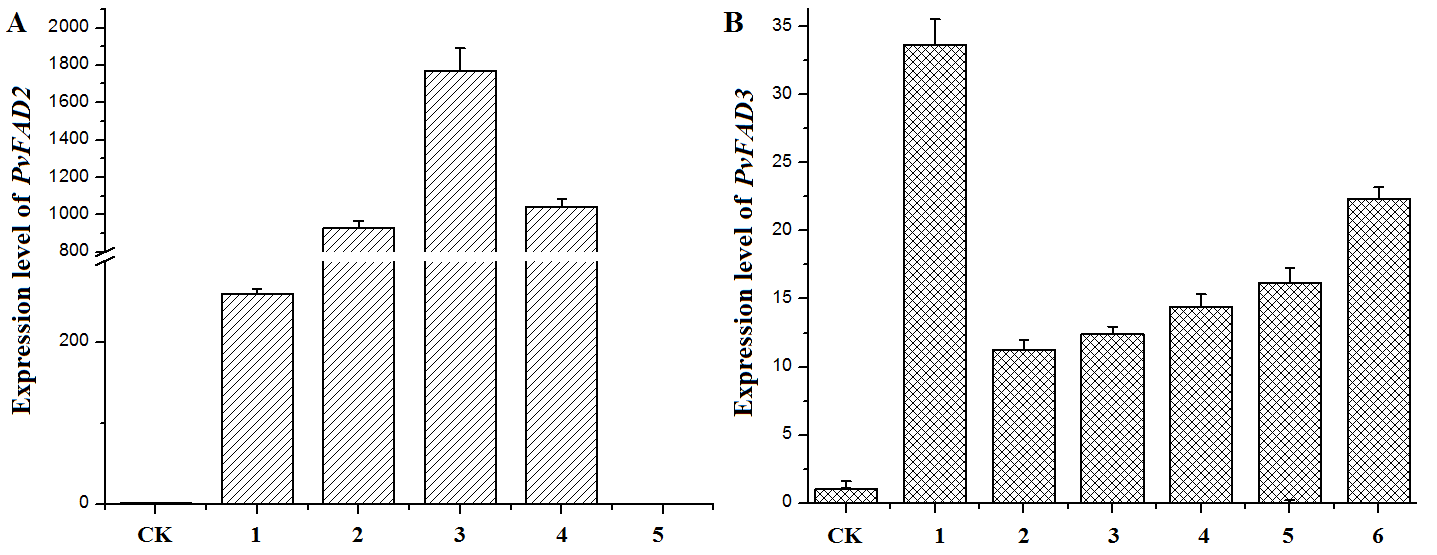

Supplement: Figure S2 — (A) PvFAD2 expression level in five transformed plants. (B) PvFAD3 expression level in six transformed plants. [file peerj-08-9169-s002.png]
