# Supplementary material for: Isolation and functional analyses of PvFAD2 and PvFAD3 involved in the biosynthesis of polyunsaturated fatty acids from Sacha Inchi (Plukenetia volubilis)
Source: PeerJ. 2020 May 26;8:e9169. doi: 10.7717/peerj.9169 (PMC7315619; doi:10.7717/peerj.9169)
Supplement: Table S1 [file peerj-08-9169-s003.pdf]

Table S1. Primers used in this study.

Unigenes	Sequences (5'-3')	Purpose
PvFAD2-F	ATGGGTGCTGGTGGCAGAATGTC	gene clone
PvFAD2-R	CGCGGTCAAAACTTGTTTTTGTACC	gene clone
PvFAD3-F1	GTGGTCAAGGAGTCCAGGCAAAGAAGGT	3' RACE
PvFAD3-F2	ACCCCAAACGAAAGGAGAGCAGTGATGA	3' RACE
PvFAD3-R1	GTGAACCTTCTTTGCCTGGACTCCTTGA	5' RACE
PvFAD3-R2	GCAAGCAAAGGGAGAGGCAGCGTGA	5' RACE
PvFAD3-F	CTGCTCTAGCTCTTCCTCTGTCTCT	Full-length
PvFAD3-R	TGCTACATGACTGTCTGTATCCTGT	Full-length
GFP-FAD2F	acgagctgtacaagcctaggATGGGTGCTGGTGGCAGAAT	Subcellular Localization
GFP-FAD2R	gtcacctgtaattcacacgtgATCAAAACTTGTTTTTGTACCAG	Subcellular Localization
GFP-FAD3F	acgagctgtacaagcctaggATGCAGACCATGGATATTTCC	Subcellular Localization
GFP-FAD3R	gtcacctgtaattcacacgtgTTAAACTAACTTGGTTTTTGCC	Subcellular Localization
Napin- FAD2F	GCTCTAGAATGGGTGCTGGTGGCAGAAT	Pant expression
Napin- FAD2R	AAAACTGCAGTCAAAACTTGTTTTTGTACCAGAAG	Pant expression
Napin- FAD3F	GCTCTAGAATGCAGACCATGGATATTTC	Pant expression
Napin- FAD3R	AAAACTGCAGTTAAACTAACTTGGTTTTTGCCAA	Pant expression
P426-PvFAD2F	gattctagaactagtggatccATGGGTGCTGGTGGCAGAAT	Yeast expression
P426-PvFAD2R	cgataagcttgatatcgaattcATCAAAACTTGTTTTTGTACCAG	Yeast expression
P426-PvFAD3F	gattctagaactagtggatccATGCAGACCATGGATATTTCC	Yeast expression
P426-PvFAD3R	cgataagcttgatatcgaattcTTAAACTAACTTGGTTTTTGCC	Yeast expression
P426-F	GGTTGAAACCAGTTCCCTGA	Sequence
P426-R	GGTTGAAACCAGTTCCCTGA	Sequence
qRT-FAD2F	TGACGATGTGTTGAAGCGAG	qRT-PCR
qRT-FAD2R	GTGGAAGAAACTGGTGGCAA	qRT-PCR
qRT-FAD3F	GACCCTAGCAATCCTCCTCC	qRT-PCR
qRT-FAD3R	GCACAATGAATCGAAGCAAAGA	qRT-PCR
Actin-F	AAGGGATGCGAGGATGGA	qRT-PCR
Actin-R	CAAGGAAATCACCGCTTTGG	qRT-PCR
