# Supplementary material for: Isolation and functional analyses of PvFAD2 and PvFAD3 involved in the biosynthesis of polyunsaturated fatty acids from Sacha Inchi (Plukenetia volubilis)
Source: PeerJ. 2020 May 26;8:e9169. doi: 10.7717/peerj.9169 (PMC7315619; doi:10.7717/peerj.9169)
Supplement: Table S2 [file peerj-08-9169-s004.pdf]

Table S2. FAD proteins used in sequence alignment and phylogenetic analysis.

**Species**	**Lable**	**Acession Number**	**Per. Ident**	**E value**
Arabidopsis thaliana	AtFAD8	AT5G05580		
Arabidopsis thaliana	AtFAD2	AAA32782.1	75%	0.0
Arabidopsis thaliana	AtFAD3	BAA05514.1	70.78%	0.0
Arabidopsis thaliana	AtFAD7	AT3G11170		
Arabidopsis thaliana	AtFAD6	AAA92800.1		
Oryza sativa	OsFAD3	BAA11397	68.44%	1e-175
Oryza sativa	OsFAD2	ACN87220.1	69.13%	0.0
Oryza sativa	OsFAD8	BAE79785		
Oryza sativa	OsFAD6	BAD09897		
Oryza sativa	OsFAD7	BAE79784.1		
Zea mays	ZmFAD2	ABF50053.1	69.31%	0.0
Zea mays	ZmFAD7	BAA22441.1		
Zea mays	ZmFAD8	BAA22442.1		
Zea mays	ZmFAD6	DAA48913		
Zea mays	ZmFAD3	NP_001149938	71.51%	4e-173
Brassica napus	BnFAD3	AAA61775.1	72.01%	0.0
Brassica napus	BnFAD2	ACP39502.1	77.66%	0.0
Brassica napus	BnFAD6	AAT65203		
Brassica napus	BnFAD7	ACS26170		
Brassica napus	BnFAD8	ACS26172		
Glycine max	GmFAD3A	AAO24263.1	75.50%	0.0
Glycine max	GmFAD3B	ABV00679.1	73.89%	0.0
Glycine max	GmFAD3C	ABV00681.1	77.31%	0.0
Glycine max	GmFAD2-2	AAB00860.1	80.73%	0.0
Glycine max	GmFAD2-1	AAB00859.1	81.25%	0.0
Glycine max	GmFAD6	AAA50158.1		
Glycine max	GmFAD7-1	ACS15381.1		
Glycine max	GmFAD7-2	ACF19424.1		
Medicago truncatula	MtFAD2-1	Medtr1g054035.1	76.36%	0.0
Medicago truncatula	MtFAD2-2	Medtr1g111880.1	67.45%	0.0
Medicago truncatula	MtFAD2-3	Medtr7g093200.1	77.40%	0.0
Medicago truncatula	MtFAD3-1	Medtr3g464330.1	72.30%	0.0
Medicago truncatula	MtFAD3-2	Medtr5g071170.1	71.77%	0.0
Medicago truncatula	MtFAD7-1	Medtr4g015630.1		
Medicago truncatula	MtFAD7-2	Medtr7g068780.1		
Medicago truncatula	MtFAD6	Medtr4g130980.1		
Solanum lycopersicum	SlFAD2	XP_004228665	75.26%	0.0
Solanum lycopersicum	SlFAD3	ABX24525	73.26%	0.0
Solanum lycopersicum	SlFAD6	XP_004242585		
Solanum lycopersicum	SlFAD7	NP_001234592		

Solanum lycopersicum	SlFAD3	ABX24525.1	73.26%	0.0
Nicotiana tabacum	NtFAD2	AAT72296	76.82%	0.0
Nicotiana tabacum	NtFAD6	AIA22326		
Nicotiana tabacum	NtFAD7	AIA22325		
Nicotiana tabacum	NtFAD3	BAA05515.1	69.55%	0.0
Olea europaea	OeFAD3	ABG88130.2	72.38%	0.0
Olea europaea	OeFAD2-2	AAW63041.1	76.04%	0.0
Olea europaea	OeFAD2-1	AAW63040.1	78.65%	0.0
Olea europaea	OeFAD6	AAW63039.1		
Olea europaea	OeFAD7	ABG88131.1		
Olea europaea	OeFAD8	XP_022887446.1		
Jatropha curcas	JcFAD3	ABX82798.1	79.30%	0.0
Jatropha curcas	JcFAD2	ACR15954.1	75.78%	0.0
Jatropha curcas	JcFAD7	ABE72960.1		
Jatropha curcas	JcFAD8	ABU96743.1		
Ricinus communis	RcFAD7	AAA73511.1		
Ricinus communis	RcFAD6	XP_002532960.1		
Ricinus communis	RcFAD2	ABK59093.1	86.49%	0.0
Ricinus communis	RcFAD3	EEF36775.1	87.07%	0.0
Vernicia fordii	VfFAD3	AF047172.2	79.72%	0.0
Perilla frutescens	PfFAD3A	AAL36934.1	69.52%	0.0
Perilla frutescens	PfFAD3B	AAD15744.1	71.79%	0.0
Triticum aestivum	TaFAD3	BAA28358.1	71.55%	0.0
Triticum aestivum	TaFAD7	BAA07785.3		
Brachypodium distachyon	BdFAD2-1	XP_010231291.2	68.62%	0.0
Brachypodium distachyon	BdFAD2-2	XP_003570235.1	68.80%	0.0
Brachypodium distachyon	BdFAD8	XP_003558157.1		
Brachypodium distachyon	BdFAD7	XP_003562400.1		
Brachypodium distachyon	BdFAD3	XP_003577162.1	68.75%	6E-179
Brachypodium distachyon	BdFAD6	XP_003574539.1		
Linum usitatissimum	LuFAD3A	ABA02172.1	93%	0.0
Linum usitatissimum	LuFAD3B	ABA02173.1	74.87%	0.0
